# Environmental conflicts and defenders: A global overview

**DOI:** 10.1016/j.gloenvcha.2020.102104

**Published:** 2020-07

**Authors:** Arnim Scheidel, Daniela Del Bene, Juan Liu, Grettel Navas, Sara Mingorría, Federico Demaria, Sofía Avila, Brototi Roy, Irmak Ertör, Leah Temper, Joan Martínez-Alier

**Affiliations:** a Institut de Ciència i Tecnologia Ambientals (ICTA-UAB), Universitat Autònoma de Barcelona, Spain; b College of Humanities and Development Studies, China Agricultural University, Beijing, China; c The Ataturk Institute for Modern Turkish History, Bogazici University, Istanbul, Turkey; d Department of Natural Resource Sciences, McGill University, Canada

**Keywords:** Environmental justice, Environmentalism of the poor, Environmental conflicts, Sustainability, Statistical political ecology, EJAtlas

## Abstract

Recent research and policies recognize the importance of environmental defenders for global sustainability and emphasize their need for protection against violence and repression. However, effective support may benefit from a more systematic understanding of the underlying environmental conflicts, as well as from better knowledge on the factors that enable environmental defenders to mobilize successfully. We have created the global Environmental Justice Atlas to address this knowledge gap. Here we present a large-n analysis of 2743 cases that sheds light on the characteristics of environmental conflicts and the environmental defenders involved, as well as on successful mobilization strategies. We find that bottom-up mobilizations for more sustainable and socially just uses of the environment occur worldwide across all income groups, testifying to the global existence of various forms of grassroots environmentalism as a promising force for sustainability. Environmental defenders are frequently members of vulnerable groups who employ largely non-violent protest forms. In 11% of cases globally, they contributed to halt environmentally destructive and socially conflictive projects, defending the environment and livelihoods. Combining strategies of preventive mobilization, protest diversification and litigation can increase this success rate significantly to up to 27%. However, defenders face globally also high rates of criminalization (20% of cases), physical violence (18%), and assassinations (13%), which significantly increase when Indigenous people are involved. Our results call for targeted actions to enhance the conditions enabling successful mobilizations, and for specific support for Indigenous environmental defenders.

## Highlights

• Support of environmental defenders requires better understanding of environmental conflicts.
• Environmental defenders employ largely non-violent protest forms.
• Indigenous environmental defenders face significantly higher rates of violence.
• Combining preventive mobilization, tactical diversity and litigation increases activists’ success.
• Global grassroots environmentalism is a promising force for sustainability.

## 1 Introduction

Environmental defenders are individuals and collectives who protect the environment and protest unjust and unsustainable resource uses because of social and environmental reasons. They may include Indigenous people, peasants or fisherfolks whose lives and livelihoods may be threatened by environmental change or dispossession, as well as environmental activists, social movements, journalists, or any other who actively defend the environment because degradation has reached for them unacceptable levels (Butt et al., 2019, Ghazoul and Kleinschroth, 2018, UNEP, 2018). The United Nations (UN) Human Rights Council has unanimously recognized the vital role of environmental defenders for environmental protection and sustainability (UN, 2019). While this formal recognition of the role of environmental defenders for sustainability is recent, already previous research has highlighted how civil society groups and grassroots movements shape the politics and practices of resource use, frequently towards positive social and ecological outcomes (e.g. Bebbington et al., 2008, Escobar, 1998, Guha and Martinez-Alier, 1997, Kenney-Lazar et al., 2018, Martinez-Alier, 2002, Scheidel et al., 2018, Villamayor-Tomas and García-López, 2018).

Such movements in defense of nature and equitable resource use are a promising force for global sustainability and just environmental futures (Nagendra, 2018, Temper et al., 2018b). Yet their activism comes at a heavy cost to both life and limb. Global Witness (2019) reported that 164 environmental defenders were murdered in 2018. The trend of annually recorded killings has generally increased over the last fifteen years (Butt et al., 2019). Defenders face not only murder and physical violence, but also severe environmental, health, and cultural impacts (Navas et al., 2018), as well as social stigma, such as being accused to act on behalf of malevolent foreign interests (Dupuy et al., 2016). The urgency of supporting and protecting defenders from violence and repression is therefore high (Knox, 2015, Tanner, 2011). While activists continue to resort to protest as a legitimate way to seek redress (Hanna et al., 2016), the UN has put forward policy frameworks to promote greater protection of environmental defenders (UN, 2018, UNEP, 2018).

However, support to environmental defenders may benefit from a more systematic understanding of the underlying environmental conflicts (Ghazoul and Kleinschroth, 2018), as well as from better knowledge on the factors that enable affected groups to mobilize successfully for environmental justice (Zabala, 2019). Awareness about killings of environmental defenders has increased substantially with Global Witness’ annual reports and recent analyses of their database (e.g. Butt et al., 2019, Middeldorp and Le Billon, 2019). Yet a global analysis of the causes and characteristics of the underlying environmental conflicts and the protest forms employed by environmental activists has been lacking. While environmental conflict studies are numerous in the field of political ecology, they frequently are limited to local or national case studies (Le Billon, 2015). Larger statistical analyses involving up to several hundred cases have become available only recently (e.g. Del Bene et al., 2018, Gerber, 2011, Haslam and Ary Tanimoune, 2016, Jeffords and Thompson, 2016, Martinez-Alier et al., 2016a, Pérez-Rincón et al., 2019). Comparative analysis of the conditions leading to success for environmental movements have been rare (see however Aydin et al., 2017, Bebbington et al., 2008, Hess and Satcher, 2019) and large global analyses have not been done at all.

In this paper, we aim to address this research gap by providing a global overview of environmental conflicts and mobilizations by environmental defenders. Our study is an analysis of the Environmental Justice Atlas database (EJAtlas, www.ejatlas.org), which we created in 2011 to foster systematic and comparative research on environmental conflicts (Temper et al., 2018a, Temper et al., 2015). We understand environmental conflicts as social conflicts over the environment that manifest through mobilizations by individuals or groups in response to perceived environmental threats with detrimental social impacts. The EJAtlas documents such conflicts in a standardized manner, based on the integration of different information sources. The extensive collaborative process has involved so far several hundred individuals and organizations worldwide. With about 3100 cases registered by April 2020, the EJAtlas has become the largest global inventory of environmental conflicts that documents also the claims and actions of involved environmental defenders.

In an effort to advance statistical political ecology, we present here the largest analysis of environmental conflicts up to date, based on 2743 recent, visible, and previously documented cases registered in the EJAtlas. Through descriptive statistics we provide a global perspective on i) where which types of environmental conflicts occur, ii) the characteristics of involved environmental defenders and how they mobilize successfully for environmental justice, and iii) important positive and negative conflict outcomes for environmental defenders. We focus not only on global rates of murder, but also on the criminalization of dissent and physical violence against activists, and how these incidences change when Indigenous people are involved in mobilizations.

We find bottom-up mobilizations for more just and sustainable uses of the environment to occur globally, testifying to the important role that diverse forms of grassroots activism play for sustainability. Environmental defenders are often comprised of vulnerable groups, acting frequently in collectives and employing largely non-violent protest forms. When Indigenous people are involved in such mobilizations, protesters face significantly higher rates of violence. Yet, mobilizations bring also important successes for environmental movements and defenders. In 11% of cases globally, protesters contributed to halt environmentally destructive and socially conflictive projects. Combining strategies of preventive mobilization, diversification of protest and litigation can significantly increase this success rate to up to 27%. These findings have direct implications for enhanced support of environmental defenders that we address in the concluding section.

## 2 Theoretical background

### 2.1 Environmental conflict research

Environmental conflicts can be broadly defined as social conflicts related to the environment. They differ, but frequently overlap, with other types of conflicts on gender, class, territory, or identity (Flint, 2005). Conflicts over natural resources have always been part of human history, for instance, *“the idea that wars are associated with resources is probably as old as war itself”* (Le Billon, 2012, p. 9). Research orientation lies often on violent and armed conflicts, although there is a wider range beyond those involving overt violence. Scholars have studied environmental conflicts from different angles and disciplines, addressing the causes, the actors and their motivations, the forms of mobilization, the outcomes, and their multiple impacts within different contexts (for a review, see Le Billon, 2015).

A prevalent argument has been that environmental conflicts are largely due to poverty or resource scarcities, which can be demand-induced, supply-induced, or structural (Homer Dixon, 1999). This implies that the occurrence and intensity of conflicts would increase as resources become scarcer, or if resources have been overused, depleted, or degraded to a certain threshold, environmental conflicts would exacerbate. In response to this frequently apolitical perspective on conflict causes, the field of political ecology emerged as a radical critique in the 1970–80s, coined by cultural ecologists, anthropologists and geographers (Blaikie and Brookfield, 1987, Watts and Peets, 2004, Peluso and Watts, 2001, Robbins, 2012). Political ecology aims to provide more nuanced analyses of power relations in environmental conflicts by departing from the *“neo-Malthusian assumptions, reductionist and essentializing character”* (Le Billon, 2015, p. 603) of the studies that primary focus on scarcity as conflict driver.

Political ecologists recognize that scarcity or abundance of resources are relative social constructs (Kallis, 2019). The transformation from ‘nature’ into a ‘resource’ is a historical process of social construction, which is related to human desires, needs and practices, and the conditions, means and forces of production (Harvey, 1996). The study of environmental conflicts sheds light on who has the power to decide about, control and allocate environmental benefits and burdens, which includes issues of distribution, access rights, and the division of labor (Robbins, 2012). Martínez-Alier and O’Connor (1996) termed such struggles over the distribution of environmental benefits and burdens, *ecological distribution conflicts*. In contrast to *economic distribution conflicts*, ecological distribution conflicts do not arise over economic costs and benefits, or being linked to profits, salaries, or prices between sellers and buyers over commodities, but are conflicts that arise over the unfair distribution of environmental ‘goods’, such as clean water and air, or access to fertile land, and ‘bads’ such as exposure to pollution, as well as risks and threats to health, livelihoods, social and cultural identities.

Studies on ecological distribution conflicts have frequently highlighted both social and biophysical dimensions of conflicts. Martinez-Alier et al. (2010) pointed to the different valuation languages apparent in environmental conflicts, reflecting incommensurable worldviews, values, and priorities of different actors. But also, the *social metabolism*, that is, the appropriation, transformation, and disposal of energy and material resources by societies, is considered a relevant conflict driver (Muradian et al., 2012). This view stems from the parallel development of the field of ecological economics, which poses that industrial economies are entropic, and not circular (Georgescu-Roegen, 1971, Haas et al., 2015). From this perspective, even a non-growing industrial economy would need a continuous supply of natural resources from the *commodity extraction frontiers* (Moore, 2000) and dispose waste to maintain itself. Consequently, this may trigger the appropriation of natural resources from other (customary) users. The growing problems of waste disposal and contamination indicate that powerful actors are able to reap the benefits from environmental goods while shifting environmental burdens to marginalized or poorer actors (Demaria and D’Alisa, 2013). By focusing explicitly on the relation of conflicts to commodities and resource use sectors, the EJAtlas and this study builds on the tradition of ecological distribution conflicts research.

Most research on environmental conflicts are site-specific case studies at the local level, or sometimes at national, regional, or sectoral scales (e.g. Amengual, 2018, Bebbington et al., 2013, Urkidi, 2010, Veuthey and Gerber, 2012, Yang and Ho, 2018). Larger comparative studies and statistical approaches have become available only recently (e.g. Gerber, 2011, Haslam et al., 2018, Haslam and Ary Tanimoune, 2016, Jeffords and Thompson, 2016) and offer new avenues to the recent calls to expand methodological plurality in political ecology (Zimmerer, 2015). Also the EJAtlas represents a new research tool: it enables standardized data collection on environmental conflicts worldwide in order to move towards a more systematic understanding of environmental conflicts (Temper et al., 2018a).

The first studies that used the EJAtlas as a novel database were published in 2015 (Latorre et al., 2015, Martinez-Alier et al., 2016a, Temper et al., 2015). A recent special issue further consolidated its use for comparative political ecology (Temper et al., 2018a). These studies employed in their analysis up to a few hundred cases and focused mainly on regional trends, such as environmental conflicts in Andean countries (Pérez-Rincón et al., 2019), sectoral dynamics, such as conflicts over wind power (Avila, 2018), dams (Del Bene et al., 2018), or mining (Aydin et al., 2017), or specific thematic concerns, such as multidimensional violence in Central American conflicts (Navas et al., 2018). The only study employing a global dataset of 1357 EJAtlas cases was published by Martinez-Alier et al. (2016b), and provided some preliminary statistics on the involved actors and mobilization forms, while focusing further on qualitative aspects, such as a description of the protest vocabulary used by environmental justice movements. Since then, the number of registered conflicts has more than doubled. With an analysis of 2743 conflicts, this article is by far the largest study using the EJAtlas data. It provides entirely new analyses of environmental conflicts in relation to sectors and income groups, actors and their successful protests forms, and key positive and negative conflict outcomes and their association with Indigenous and non-indigenous mobilizations.

In this regard, the EJAtlas has not only enabled multi-sites comparative studies with larger samples in geographical, sectoral, or thematical terms. The EJAtlas also expands the research scale of political ecology to a global level to advance a comparative statistical political ecology. Without dismissing the importance and richness of in-depth case study and other qualitative methods, we argue that such a broad comparative view can reveal global patterns that are relevant for a more systematic understanding of the characteristics of environmental conflicts worldwide, the actors involved, and their successful mobilization forms.

### 2.2 Environmental defenders: terms and concepts

Among the key actors in environmental conflicts are those that defend the environment against negative social or ecological impacts, because their lives and livelihoods depend on healthy ecosystems, or because of other directly related social or environmental reasons. Such actors have been termed *environmental defenders* in media, civil society reports (e.g. Global Witness, 2019, Global Witness, 2014), academia (Butt et al., 2019, Knox, 2015, Martinez-Alier et al., 2016b, Middeldorp and Le Billon, 2019, Tanner, 2011), and recently also in international human rights policies. The UN Environment Programme refers to environmental human rights defenders as “*anyone (including groups of people and women human rights defenders) who is defending environmental rights, including constitutional rights to a clean and healthy environment, when the exercise of those rights is being threatened whether or not they self-identify as human rights defenders. Many environmental defenders engage in their activities through sheer necessity”* (UNEP, 2018). This may include Indigenous people, peasants, fisherfolks, environmental activists, social movements, journalists, or any other people concerned over adverse corporate or state-driven resource uses and related environmental change.

In protesting and mobilizing against the exploitation of nature, environmental defenders frequently serve a larger purpose of environmental protection, even though their actions are not always framed as such (Ghazoul and Kleinschroth, 2018). The UN Human Rights Council (2019) asserts that there can be no environmental protection without recognition and respect for environmental defenders. The contributions of environmental activists to sustainability have also been highlighted in the academic literature on environmental conflicts and transformations towards sustainability (e.g. Nagendra, 2018, Scheidel et al., 2018, Temper et al., 2018b).

While the term *environmental defenders* and the attention given to it is recent, it relates to previous debates.^1^ Guha and Martinez-Alier (1997) introduced the concept of an *environmentalism of the poor* as early as in the 1980s to describe the environmental protection actions by poor people who were struggling against the degradation of the environment upon which their livelihood depended. Similarly, Indian scholars Gadgil and Guha (1995) called them *ecosystem people*, highlighting how many rural dwellers rely on healthy ecosystems. The idea of an environmentalism of the poor emphasized the material and social interest in the environment as a livelihood source for marginalized groups in rural areas in the global South. It questioned the theory that only rich people would defend the environment because they have their needs covered and thus can prioritize ecological actions (Bell, 2020, Martinez-Alier, 2002).

In the same period that the notion of an environmentalism of the poor was put forward, the idea of *environmental justice,* and a strong social movement supporting it, was born in the United States during the struggles against waste dumping in North Carolina in 1982. Environmental justice was defined and developed by civil rights activists and members of Christian churches as well as sociologist Robert Bullard (Bullard, 1994, Bullard, 1990). Their protests began to make a connection between racism and social injustices and the negative environmental impacts suffered by people of color in urban or peri-urban areas in the United States. The neighborhoods where African Americans lived were the most contaminated as most landfills were allocated there.

By taking a global view, Anguelovski and Martínez Alier (2014) argued that, despite the diversity of actors and their different origins, the overall concerns of environmental justice movements and the environmentalism of the poor frequently converge over aims to reassert customary practices and to protect lands and livelihoods from adverse environmental change. Here we take such a global perspective and use the term environmental defenders to refer to any individuals, civil society groups and social movements that mobilize against unsustainable or socially unjust uses of the environment, no matter if they are from the global North or South, or whether social or ecological reasons are their primary motives.

### 2.3 Violence in environmental conflicts

The assassination of environmental defenders is the highest and most visible expression of direct violence, but it is not the only one appearing in environmental conflicts (Navas et al., 2018). *Structural violence* is understood as a process that refers to the violence ingrained in the social, political, and economic structures, producing discrimination or social inequality (Farmer, 2004). Cultural violence refers to how cultural elements (i.e., language, religion, or ideology) are used to legitimize the former forms of violence (Galtung, 1990). Slow violence points out the daily and long-lasting violence, caused, for instance, by the increasing and cumulative effects of daily exposure of communities to contamination by extractive industries such as mining (Nixon, 2011).

Given these manifold forms of violence, Navas et al. (2018) called for a multidimensional approach to violence in environmental conflict research. While we recognize the importance to address the subtler forms of violence, these are also more difficult to be tracked and assessed at the global level. Based on the data provided by the EJAtlas, we focus in this study on three aspects of violence: a) assassinations, b) physical violence against activists and c) criminalization of environmental defenders. For a definition of these terms, and all other variables analyzed in this study, see Appendix A.

## 3 Methods

### 3.1 Overview

The study presents a quantitative analysis of 2743 cases of environmental conflicts, the characteristics of the involved environmental defenders, successful mobilization strategies, and positive and negative conflict outcomes from the perspective of environmental defenders. The cases were documented based on secondary sources and were coded in a standardized manner through the EJAtlas.

### 3.2 The Environmental Justice Atlas (EJAtlas)

The EJAtlas was created in 2011 through a collaborative process between academics and civil society groups (Temper and Del Bene, 2016). Among the aims of establishing the EJAtlas was to advance and expand political ecology by going beyond case study research and moving towards large comparative and statistical analyses (Temper et al., 2018a). Today, it constitutes the largest global database on environmental conflicts and the involved actors mobilizing for environmental justice. For further information on the EJAtlas rationale, see Temper et al. (2015).

### 3.3 Unit of analysis and case documentation

The unit of analysis of the documented cases is an environmental conflict provoked by a specific state- or corporate-driven resource use project (e.g. a hydroelectric dam, or a mine) due to perceived risks and negative socio-environmental impacts triggering mobilizations. Perceived threats may include social and environmental impacts that were either documented or directly noted by local groups in the absence of formal assessments, or anticipated risks severe enough to trigger conflict. The latter is, for instance, frequently the case for nuclear power plants. Conflict cases are documented in a standardized form that includes information on general characteristics (location, relevant background information), project details, companies, finance institutions and government actors involved, visible and potential social and environmental impacts, actors mobilizing to defend the environment, forms of mobilizations used, conflict outcomes, and references to relevant legislation, academic research, videos, and other media. Note that this focus is broader and different than Global Witness’ database on environmental defenders. While the latter principally focuses on events of killings as the unit of analysis, the EJAtlas focuses on the underlying environmental conflict and protest dynamics as the unit of analysis, whereas one conflict could involve several assassinations.

Information on conflict events is coded and provided also qualitatively in the EJAtlas as descriptive texts. Conflicts are mapped by economic and resource use sectors provoking the conflict, covering ten main categories: biomass and land use, conservation, energy and climate, industries, infrastructures, mining, nuclear, tourism, waste management, and water management (for definitions, see Appendix A). The socio-environmental concerns in conflicts frequently overlap across various categories, e.g. a mine may cause also land conflicts over land acquisition for the mining concession. In such cases, conflicts are categorized in one of the ten mutually exclusive main categories based on the sector causing the conflict, which in the previous example would be mining. However, the ten main categories can be complemented in the EJAtlas by indicating 52 mutually non-exclusive sub-categories.

### 3.4 Data collection, validation and quality checks

The scarcity of grounded data is a major challenge for a better understanding of the local dynamics of environmental conflicts. Under such circumstances, the use of local and non-academic knowledge sources is a valuable approach to overcome knowledge gaps (Couzin, 2007, Gerber, 2011, Huntington, 2011). The use of newspaper accounts on conflict and mobilization events is also a common practice in social movement studies, despite limitations on potential coverage bias (for a thorough discussion and justification see Earl et al. (2004)). The EJAtlas has made a substantial effort in facilitating data gathering from various sources on a global scale to bring local knowledge to environmental conflicts research (Temper et al., 2015, Temper and Del Bene, 2016).

Case data are collected through a collaborative process among academics and civil society actors, whereas individuals (e.g. academics, journalists, environmental activists and other knowledgeable persons) and collectives (e.g. local associations, non-governmental organizations, academic groups, and other collectives) must first register in the EJAtlas as contributors. Once registered, collaborators can identify environmental conflict cases, and provide information and secondary sources on the conflict events. Data gathering over the past eight years has involved several hundred collaborators.

The EJAtlas documents only cases that are verifiable through secondary sources, published previously elsewhere. Sources include academic papers, newspaper articles, lawsuits, formal complaints and other legal documents, civil society reports, and other sources. Case documentation is coordinated, and the quality of the provided information is reviewed, cross-checked and validated by a permanent team located at Universitat Autònoma de Barcelona (UAB), who also counts on external experts’ support if necessary. The same team also assures consistency and completeness in the coding of reported conflict events. All conflict cases analyzed here and the way they have been coded can be looked up online at www.ejatlas.org.

### 3.5 EJAtlas dataset and limitations

The resulting EJAtlas dataset is a large convenience sample of recent and previously documented conflicts from an unknown total number of environmental conflicts worldwide. Therefore, the dataset is statistically not representative globally; the shown frequencies and associations of observations reflect the distributions within the EJAtlas dataset. Similar limitations apply also to several other global conflict datasets, such as Global Witness’ data on killings of environmental defenders, or the NGO GRAIN’s database on land grabbing conflicts that was used by research institutions for describing global land grab characteristics (e.g. World Bank, 2010).

This is an important caveat that has several implications for interpreting EJAtlas data. First, some regions such as parts of Russia and Mongolia, Central Asia, and Central Africa have limited coverage in the EJAtlas. This may result in the underrepresentation of actors and mobilizations forms common in these areas, such as conflicts involving pastoralists (Fratkin, 1997). Second, some countries are mapped in more detail than others, not necessarily because of having more conflicts, but because of better data availability. This limits possibilities for meaningful comparisons across countries and continents, such as whether one country has more conflicts than another. However, global country groupings by income, as defined by the World Bank, are relatively homogenously covered in terms of documented conflicts per millions of people (see Appendix A for a discussion and data). Therefore, we compare here only world income regions. Third, the EJAtlas has limited information on environmental conflicts in war zones, where confrontations may be embedded in more violent histories and contexts. Further inclusion of environmental conflicts from such areas could lead to an increase of violent events reported in the dataset, both against and by protesters.

Despite these limitations, the EJAtlas dataset represents currently the most extensive global sample available on environmental conflicts. Therefore, it allows for new insights from a broad comparative perspective that has not been possible before. Where applicable, we encourage the comparison of results with other databases to further assess the strength of derived findings.

### 3.6 Statistical analysis

The sample analyzed here (n = 2743) includes all conflicts that were registered, reviewed, and approved for publication on the EJAtlas since its inception in 2011 and until March 26^th^, 2019. These are predominantly recent conflicts: 95% of the 2743 cases began during or after 1970; 50% of the cases began during or after 2008 and reach the present (for more details, see Appendix A). We use descriptive statistics to analyze the characteristics of environmental conflicts, the environmental defenders involved, and the mobilization strategies used. Results are presented by indicating the frequency of observations, percentages of the total sample, and confidence intervals at 95%. We use Pearson’s Chi-square tests of independence to examine the associations between selected conflict outcomes (project cancellation, assassinations, violence against activists, criminalization) and different sectors (main EJAtlas categories), mobilization strategies (timing of mobilizations, legal actions, and protest diversification) and actors (involvement of Indigenous groups in mobilizations). Reported p-values are two-tailed. The significance level was set at 5%. All data used in this article are provided as data tables in Appendix A.

## 4 Environmental conflicts across world income regions

**Fig. 1:**
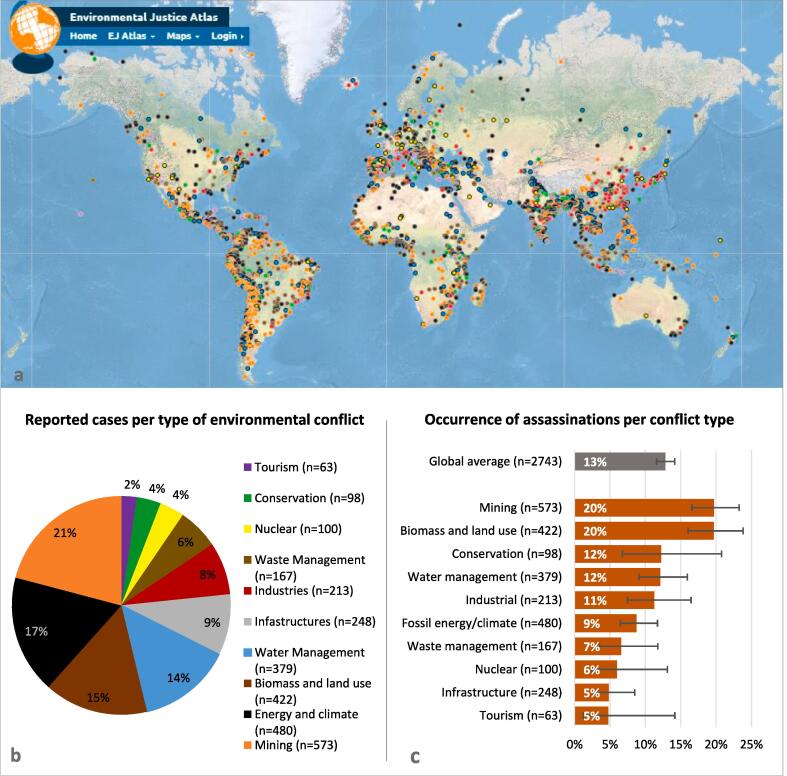
Environmental conflicts registered in the EJAtlas and occurrence of assassinations of environmental defenders across conflict types (n = 2743). a: Geographical coverage of environmental conflicts reviewed here (each dot represents one case). b: Types of conflicts and coverage (pie colours corresponds to the colour of the cases shown in the map). c: Occurrence of assassinations of environmental defenders per conflict type. Error bars are 95% CIs.

Environmental conflicts are driven by a range of economic activities related to resource extraction, processing, and waste disposal. Of the 2743 cases documented in the EJAtlas and analyzed here (Fig. 1a), the most frequently reported sectors are the mining sector (21% of all cases), the (fossil) energy sector (17%), biomass and land uses (15%), and water management (14%) such as dams (Fig. 1b). These activities concentrated in the extractive and agrarian sectors are those which are most associated with assassinations of environmental defenders (Fig. 1c). The murder of the Cambodian forest and land activist Chut Wutty in 2012 motivated Global Witness to start the systematic documentation of killings of environmental defenders worldwide (Global Witness, 2012). The killing of Berta Cáceres in 2016, who opposed the Agua Zarca hydroelectric dam in Honduras, caused an international outcry that reinforced global efforts for better protection (Middeldorp and Le Billon, 2019). Globally, 13% of the environmental conflicts documented in the EJAtlas involve assassinations of environmental defenders (Fig. 1c).

We find mining and land conflicts, with assassinations occurring in one out of five conflict cases, significantly deadlier than other categories and the global average (Pearson χ^2^ = 77.58, df = 9, p < 0.001), which is consistent with Global Witness data (Butt et al., 2019). Even projects aiming to enhance sustainability, such as conservation zones and renewable energy infrastructures, frequently cause conflicts over restriction of livelihood activities or enforced evictions (Avila-Calero, 2017, Brockington and Igoe, 2006, Del Bene et al., 2018, Scheidel and Sorman, 2012, Schleicher et al., 2019). Assassinations associated with the establishment of conservation zones occur in our data in one out of eight conflict cases. This points to how initiatives relevant for environmental sustainability that do not address social justice concerns can lead to severe and violent conflict.

**Fig. 2:**
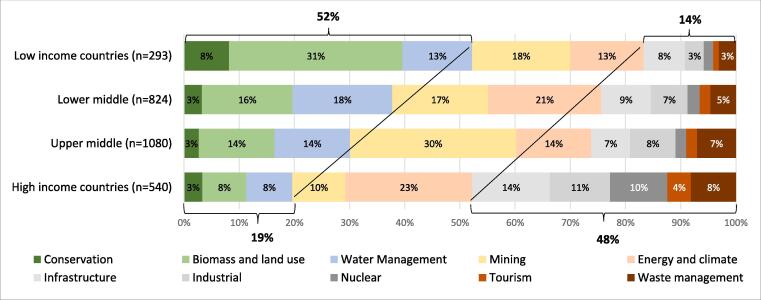
Occurrence of types of environmental conflicts across world income regions (n = 2737).

EJAtlas data show that environmental conflicts occur across all country income groups, whereas the relative prevalence of conflict types changes with economic development (Fig. 2). (For an analysis of the relation between income and killings of environmental defenders, see Jeffords and Thompson (2016)). Conflicts over conservation, biomass and land, and water management (i.e. dams), account for 52% of all cases in low-income countries, yet they account only for 19% in high-income countries. Conversely, conflicts about waste management, tourism, nuclear power, industrial zones, and other infrastructure projects account for a minor share (14%) in low-income countries but rise to almost half of all conflicts (48%) in high-income countries. Likewise, in poorer countries, most environmental conflicts recorded in the EJAtlas are rural, and as income levels per capita increase, urban and semi-urban conflicts account for an increasing share, representing up to half of all conflicts (see Appendix A, supplementary Table 4). The triggers of environmental conflicts vary thus with patterns of industrialization, urbanization, and technology use, and environmental conflicts emerge in new sectors along the lines of economic development. Muradian et al. (2012) explain this by pointing to the changing social metabolism associated with economic development, i.e. the growing demand for sources of material and energy provision, and sinks required for waste, pollution and emissions (see also Martinez-Alier et al., 2016a, Spiric, 2018). These changes fundamentally reconfigure resource extraction and use patterns and thus affect the distribution of environmental benefits and burdens across different actors and sectors.

In urban areas of middle to high-income countries, mobilizations arising in environmental conflicts are frequently termed movements for *environmental justice*, while in rural areas of low-income countries, they have been referred to as *environmentalism of the poor* (see Section 2)*.* There are certainly regional and sectoral differences between them that shape conflict dynamics, specific movement concerns, strategies, and outcomes (see Bebbington et al., 2008, Borras et al., 2018, Edelman and Borras, 2016). Yet, they commonly share overarching goals of just and sustainable resource uses, based on the reaffirmation of customary practices as well as on concrete efforts to protect their living environment from adverse change (Anguelovski and Martínez Alier, 2014). The fact that such bottom-up mobilizations for socially and environmentally more benign forms of resource uses are widely documented in the EJAtlas, across large parts of the world and among all country income groups, testifies that various forms of grassroots environmentalism exist globally. This is a promising force for sustainability and just environmental futures.

## 5 Environmental defenders and successful mobilization strategies

**Fig. 3:**
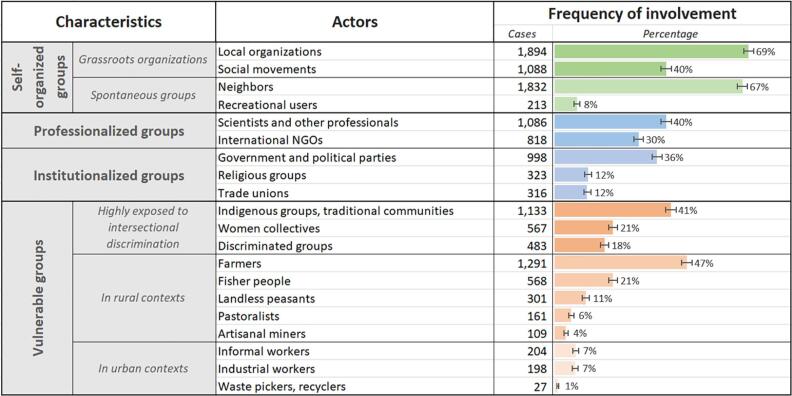
Characteristics of actors mobilizing in environmental conflicts according to the EJAtlas (n = 2743). Error bars are 95% CIs.

Environmental defenders are frequently self-organized local groups (Fig. 3), such as local associations, social movements, neighbors and recreational users, driven to action over concerns about local socio-environmental impacts. Both local organizations (involved in 69% of EJAtlas cases) and neighbors (67%) are the two most frequent actor groups mobilizing to defend their environment. While formal recognitions for environmental defenders, such as the Goldman Environmental Prize, as well as media reports and statistics of killings tend to portray the individual struggles of defenders, the high frequency of groups involved in environmental mobilizations shows the importance of collective struggle.

Institutionalized groups, such as political parties (active in 36% of cases), trade unions (12%), or religious groups (12%), appear less frequently globally, but their involvement can be decisive. Trade unions intervene in such conflicts in industrial areas to support healthy work conditions as part of a *working-class environmentalism* (Barca and Leonardi, 2018). Religious groups are important supporters, particularly in Southeast Asia and Latin America, but also in the US. For example, the United Church of Christ played a leading organizational role in the US environmental justice movement (UCC, 1987). Buddhist monks frequently shape environmental activism in Southeast Asia, where customary and sacred landscapes such as forests are threatened by state and corporate economic activities (Walter, 2007). Professional organizations and supporters, such as international NGOs (active in 30% of cases) and local scientists (active in 40% of cases) can become important allies. They may help to legitimize local claims in the media and international fora, facilitate regional and global networking, and engage in the collection of scientific evidence on risks and impacts to support movements’ claims.

Environmental defenders belong frequently to vulnerable segments of society that are disproportionally threatened by development projects and resource exploitation (cf. Blaikie et al., 1994). Many are exposed to intersectional discrimination and subject to vexed dynamics of class, ethnicity, or gender that generate both risk and inequality (Acker, 2006, Thomsen and Finley, 2019). Of these, we find in the EJAtlas that Indigenous people mobilize most frequently against damaging environmental activities, appearing in 41% of documented environmental conflicts. 47% of cases involve farmers (including Indigenous ones), underlining the need to ensure appropriate land tenure rights (FAO and CFS, 2012). 21% of EJAtlas cases highlight the role of women as leaders and claimants for feminist rights in the mobilizations, sometimes because of being disproportionally affected by environmental and health impacts (Deonandan et al., 2017, Rodriguez Acha, 2017). Many of them also face repression and killings (Martinez-Alier and Navas, 2017).

**Fig. 4:**
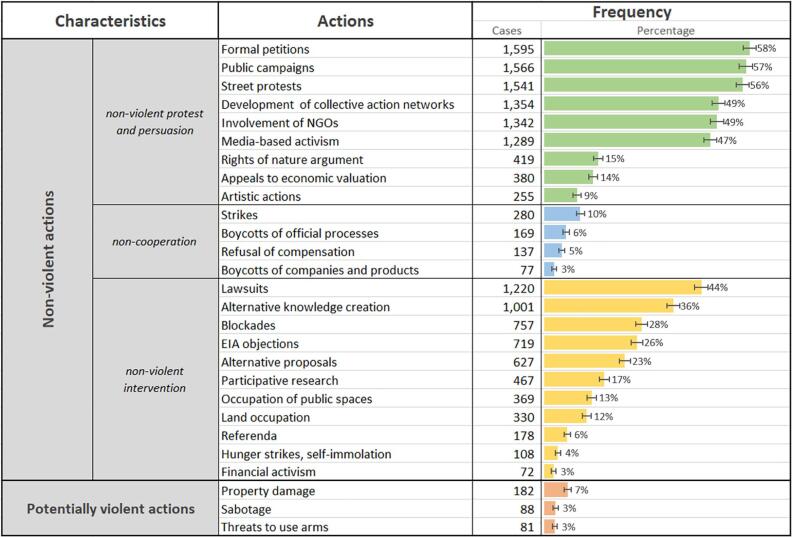
Characteristics of forms of mobilization and protest reported globally in the EJAtlas. Protest actions are clustered following Sharp (1973) (n = 2743). Error bars are 95% CIs.

Diverse forms of protest shape environmental defenders’ repertoire of contention (Fig. 4). The vast majority are non-violent actions that, following Gene Sharp (1973), we group here into acts of *non-violent protest and persuasion*, *non-cooperation*, and *non-violent intervention*. Formal petitions (reported in 58% of all cases), public campaigns (57%), and street protests (56%) are the most commonly reported forms of protest and persuasion, followed by the creation of collective action networks, involvement of NGOs, and media-based activism. Strikes, boycotts of official processes, companies, and products, or refusal of compensation payments are relevant forms of non-cooperation particularly in urban contexts, however, they appear globally only in up to 10% of all cases.

Environmental defenders use different forms of non-violent interventions. We find legal strategies such as lawsuits in almost half of all cases, and objections to environmental impact assessments (EIAs) in a quarter of all cases. Local scientists and professionals frequently support the creation of new knowledge and alternative proposals (see Conde, 2014). Reports that provide the perspective of affected communities on conflictive projects are produced in 36% of cases, while alternative project proposals are put forward in 23% of cases. More disruptive interventions such as road blockades (28%), occupation of public buildings (13%), land occupation (12%), and self-sacrifice are often employed when previous interventions were not successful (Hanna et al., 2016). Their use depends also on the political culture; for instance, 40% of cases involving hunger strikes come from India and reflect the Gandhian tradition of civil disobedience (see also Williams and Mawdsley, 2006).

Potentially violent protest actions, such as property damage, sabotage, or threats to use arms have been documented in 7%, 3%, and 3% of cases, respectively, testifying to the overwhelmingly non-violent character of defenders’ protest actions.

**Fig. 5:**
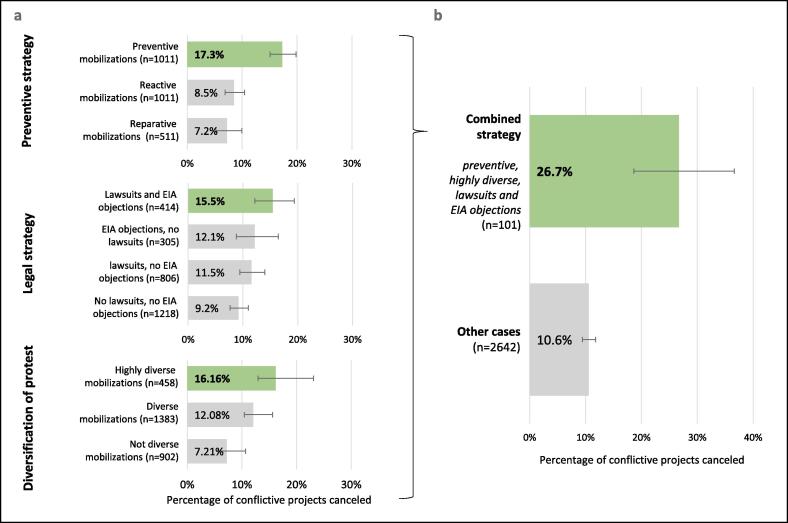
Mobilization strategies and project cancellation rates. (a) Percentage of cancellation of conflictive projects in relation to three different strategies: preventive strategy (n = 2533), legal strategy (n = 2743) and diversification of protest (n = 2743). (b) Percentage of cancellation of conflictive projects in cases with a combined strategy (preventive, highly diverse, lawsuits and EIA objections) (n = 101). Note: Highly diverse mobilizations = use of 10 or more different mobilizations forms as reported in Fig. 4; diverse mobilizations = use of 5–9 different mobilization forms; not diverse mobilizations = <5 different mobilization forms. For definition of other categories see supplementary Tables 6–9, 14. Error bars are 95% CIs.

Understanding how civil society movements mobilize successfully is important for developing effective support (Hess and Satcher, 2019, Zabala, 2019). While successes take many forms (e.g. Özkaynak et al., 2015), the cancellation of conflictive projects with adverse socio-environmental impacts is a common goal of those mobilizing and it is worth examining those protest strategies that achieve project cancellations more frequently. Here we analyze project cancellation in relation to three specific mobilization strategies: timing of mobilizations, protest diversification, and pursuit of legal actions. We acknowledge that many other factors beyond mobilization strategies influence whether success is achieved or not (e.g. political climate or movement diversity, see for instance Bebbington et al., 2008, Aydin et al., 2017). Yet the significant differences observed in conflict outcomes in relation to these strategies reveal their relevance for mobilizations (Fig. 5).

When mobilizations were preventive, undesired projects were canceled in 17% of all cases. This is about twice as much as when mobilizations occurred in reaction to project implementation, or for reparations once impacts were experienced (Pearson χ^2^ = 50.36, df = 2, p < 0.001). Besides the situated contexts of specific cases, environmental mobilizations initiated in a preventive stage usually imply better awareness of the risks and access to information, knowledge, and networks of local groups and key actors (Bondes and Johnson, 2017, Christoph Steinhardt and Wu, 2016). Features like this may come together with preventive actions, such as early campaigning, formal objections to impact assessments before projects are constructed, or alternative knowledge creation from the onset to point out neglected risks or frame alternative pathways. Furthermore, it is arguably easier to stop a project during planning phase because more leverage points exist for groups to intervene, fewer resources have been invested so that the cancellation costs are lower for state and corporate entities, and a longer timeframe for negotiating and creating alternatives is available. Recognizing the effectiveness of preventive protest has key implications for supporting environmental defenders: it points to the need to address those factors that inhibit or enable preventive mobilization.

Where protesters used more than ten different mobilization forms, projects were again more than twice as frequent to be canceled (16%) than in cases with less than five types of protest actions (7%) (Pearson χ^2^ = 26.93, df = 2, p < 0.001). Such tactical diversity is arguably beneficial because it reflects a range of skills available in the movement, it allows more people to participate in a diverse range of protest activities and thus to increase pressure on proponents of conflictive projects, and it may turn mobilizations more resilient as claimants can move between protest forms in case of repression of a particular one (Chenoweth and Stephan, 2011). Regarding legal strategies, the filing of lawsuits alone does not significantly associate with higher cancellation rates. However, when combined with formal objections to EIA, we observe a cancellation rate of 15.5% (Pearson χ^2^ = 12.87, df = 3, p < 0.01).

The most successful way to mobilize seems to not rely on a single strategy but to combine several at once (Fig. 5b). We find a significantly higher project cancellation rate of 26.7% (Pearson χ^2^ = 25.67, df = 1, p < 0.001) in those cases where mobilizations were preventive, highly diverse and took strong legal action (lawsuits and formal EIA objections). Effective support for environmental defenders should thus promote measures that enable them to pursue litigation, preventive protest and diverse mobilizations all together.

## 6 Outcomes of environmental conflicts

**Fig. 6:**
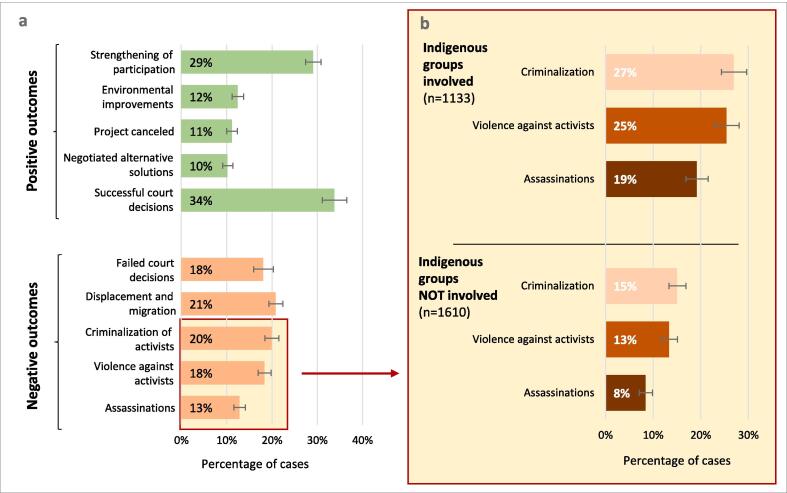
Positive and negative conflict outcomes from the perspective of environmental defenders (a) and occurrence of repressive outcomes when Indigenous groups are involved (b). n = 2743, except for cases with lawsuits (court successes and failures), where n = 1220. Error bars are 95% CIs.

Both positive and negative outcomes for environmental defenders mark environmental conflicts (Fig. 6a). One frequent positive social outcome is strengthened participation among affected people (documented in 29% of cases), including cases of increased civic engagement and participation in consultation, planning, and politics related to project development. Other positive outcomes include environmental improvements (12%), such as through the rehabilitation of degraded areas. Negotiated alternative solutions, such as negotiated reductions of conflictive land concessions to mitigate community impacts, or changes in the routes of conflictive pipelines, are reported in 10% of cases. More radical achievements from the perspective of environmental defenders are the above-discussed cancellation of conflictive projects, apparent on average in 11% of all EJAtlas cases. The struggles led by environmental defenders can thus bring important social and environmental benefits and evidence how environmental movements are important actors for sustainability.

However, for the case of renewables and conservation areas, tensions between social and ecological sustainability goals may become apparent. For instance, while locally affected groups may celebrate the cancellation of an unjust wind park, others may worry about not reaching renewable energy goals. Given that sustainability is multidimensional and multiscalar, such tensions between global environmental goals and local socio-environmental impacts are not surprising. Environmental conflicts play an important role here in fostering societal negotiation processes and the search for alternatives that are both ecologically sustainable and socially more just (Scheidel et al., 2018).

In cases where defenders were able to take legal actions, 18% of cases reported a court failure while 34% of cases a court success (some cases may have both due to multiple and overlapping proceedings). Legal successes can take many forms, such as winning demands for monetary indemnification, land restitution, recognition of customary land rights, or orders to suspend and cancel conflictive and unsustainable projects. The relatively high rate of court victories suggests that many conflictive projects do not develop in compliance with prevailing laws as well as social and environmental standards. This emphasizes the need for enhanced monitoring and accountability of corporate and state-led resource use projects and confirms the importance of improving defenders’ access to justice as an effective way of support.

Regarding negative outcomes, 21% of the 2743 cases provoke displacement, either directly caused by corporate or state-driven projects (Satiroglu and Choi, 2015), or due to the adverse effects of environmental change (Rechkemmer et al., 2016). Defenders face also physical violence (18% of all cases) and assassinations (13%). Criminalization of dissent, for instance, through imprisonment, restriction of activists' rights, or prosecution without clear charges (Moore et al., 2015), appears in 20% of cases and shows the structural violence that environmental defenders face.

Butt et al. (2019) have highlighted the role of structural factors and country contexts (i.e. rule of law, corruption) in shaping the occurrence of violence in environmental conflicts. Our data furthermore evidence that Indigenous environmental defenders are significantly more susceptible to various forms of violence (Fig. 6b). While assassinations occur in 8% of cases when Indigenous people are not involved, killings rise dramatically to 19% when Indigenous people are part of the mobilizations (Pearson χ^2^ = 68.93, df = 1, p < 0.001). With Indigenous involvement, also the occurrence of criminalization of dissent (25%) and physical violence against activists (27%) is significantly higher than in cases where Indigenous people were not involved in mobilizations (with Pearson χ^2^ = 64.65, df = 1, p < 0.001 and Pearson χ^2^ = 58.87, df = 1, p < 0.001, respectively). Indigenous populations have historically suffered from coloniality and racism (Quijano, 2000). This trend has not changed until today. Furthermore, state and corporate pressure to exploit Indigenous territories is high as they represent to the global economy some of the remaining frontiers of resource extraction. The significantly higher exposure to criminalization, violence, and assassinations underlines the urgent need to specifically support Indigenous environmental defenders.

## 7 Concluding discussion

Our results show that non-violent bottom-up mobilizations in response to adverse environmental and social impacts of economic activities and development projects occur worldwide across all income groups, testifying to the existence of various forms of grassroots environmentalism globally. This indicates a promising force for environmental sustainability and social justice, yet one that comes too often at a heavy cost of violence and repression. To enhance the protection and support of environmental defenders, we close this article by discussing some implications of our results.

First, the EJAtlas database indicates that, assassinations, physical violence and criminalization occurs significantly more often in mining and land conflicts and when Indigenous groups are involved in mobilizations. These results are consistent with Global Witness data (Butt et al., 2019) and the strong evidence for these findings emphasizes the urgent need for developing specific protection mechanisms within these sectors and particularly for Indigenous people. The role of Indigenous communities in environmental defense must be recognized and celebrated. Ongoing efforts to recognize their territories and the right to self-determination as enshrined in the United Nations Declaration on the Rights of Indigenous peoples, must be accelerated (Feiring, 2013, UN, 2008).

Second, effective support for environmental defenders should enhance the conditions that enable successful mobilizations to defend livelihoods and the environment. We found that strategies pursuing preventive mobilizations, diversification of protest, and legal actions are important to achieve positive outcomes, and particularly successful, when combined. Towards this end, access to justice must be improved beyond the development of general policy frameworks (Knox, 2015). Concrete measures could include the provision of free legal education, training, and aid, as well as monetary support to cover related expenses. Leverage points for legal interventions can be identified by tracking the legal liabilities of involved companies across the entire investment chain (Blackmore et al., 2015). This requires states and companies to enforce corporate transparency and accountability (Fox, 2007), not only where investments are made, but also in investing countries for human rights abuses committed by their corporations abroad. Transparency must also be improved in public administration, as early disclosure and knowledge about development plans, project bids and tenders are key to enable preventive mobilizations.

However, the effectiveness of such recommendations, policies and formal procedures has also limits. Environmental conflicts develop within complex political, socio-economic, and cultural contexts that do not necessarily respond to such measures. Many governments are not supportive of environmental defenders and rather seek to delegitimize them, for instance by stigmatizing them as agents of foreign influence to limit support from international actors (Matejova et al., 2018). Bottom-up protest in its manifold forms remains then a central and necessary strategy for the claims-making of affected groups, particularly when external support is constrained and when existing formal procedures, such as project safeguards, free prior informed consent, or social and environmental impact assessments are not conducted or enforced by states (Hanna et al., 2016). Diversification of protest, which we found to be highly relevant to achieve movement goals, can be enhanced through networking and sharing of knowledge about successful mobilizations. The EJAtlas, apart from opening avenues for comparative political ecology research on such themes, can be a useful resource for activists, as it documents diverse mobilization strategies and their outcomes across the globe. Furthermore, it can also be used as an advocacy map for citizens’ grievances to reach diverse actors, such as local and national government bodies as well as global media (Drozdz, 2020).

Finally, to address some of the underlying drivers, it is important to consider that environmental conflicts are embedded in global economic structures that require continuous resource extraction (Muradian et al., 2012). Our results show that environmental conflicts do not disappear with economic development but are shifted to new sectors, following the changes in resource uses. A lasting reduction of pressures on local communities’ territories as sources of resource extraction or sinks for pollution and emissions will require a substantial downscaling of the global social metabolism (Akbulut et al., 2019, Scheidel and Schaffartzik, 2019). Possible pathways to achieve this are currently being discussed, explored, and practiced in research and civil society (e.g. Escobar, 2015, Kothari et al., 2019). This process should consider the constructive potential of environmental conflicts, that is, the many ideas and proposals about alternatives put forward by environmental defenders in their effort to find more sustainable and socially just environmental futures.

## Declaration of Competing Interest

The authors declare that they have no known competing financial interests or personal relationships that could have appeared to influence the work reported in this paper.

## Footnotes

1 Other approaches not covered here have referred to this phenomenon as *subaltern environmentalism*, *livelihood ecology* or *liberation ecology* (see Martinez-Alier 2002 for an overview).
