# Supplementary material for: Environmental conflicts and defenders: A global overview
Source: Glob Environ Change. 2020 Jul;63:102104. doi: 10.1016/j.gloenvcha.2020.102104 (PMC7418451; doi:10.1016/j.gloenvcha.2020.102104)
Supplement: Supplementary data 1 [file mmc1.pdf]

Environmental conflicts and defenders: a global overview

Arnim Scheidel[a], Daniela Del Bene[a], Juan Liu[a,b*], Grettel Navas[a], Sara Mingorría[a], Federico Demaria[a], Sofía Avila[a], Brototi Roy[a], Irmak Ertör[a,c], Leah Temper[a,d], Joan Martinez-Alier[a]

[a] Institut de Ciència i Tecnologia Ambientals (ICTA), Universitat Autònoma de Barcelona (UAB), Spain
[b] College of Humanities and Development Studies, China Agricultural University, Beijing, China
[c] The Ataturk Institute for Modern Turkish History, Bogazici University, Istanbul, Turkey
[d] Department of Natural Resource Sciences, McGill University, Canada

*Corresponding author: Dr. **Juan Liu**, juanlcau@gmail.com
Institut de Ciència i Tecnologia Ambientals - Universitat Autònoma de Barcelona (ICTA-UAB).
Edifici Z 129 (ICTA-ICP). Campus de la UAB 08193 Bellaterra (Cerdanyola del Vallès). Barcelona, Spain.

DOI: https://doi.org/10.1016/j.gloenvcha.2020.102104

APPENDIX

1 SUPPLEMENTARY METHODS 1

2 SUPPLEMENTARY RESULTS 2

3 SUPPLEMENTARY DEFINITIONS 7

1 SUPPLEMENTARY METHODS

The study draws on data from the global Environmental Justice Atlas (EJAtlas, see www.ejatlas.org)[1]. Here we describe the characteristics of the EJAtlas dataset, additional data employed, and the use of data analysis software.

1.1 EJAtlas data set description

The data set analyzed in this study has a total of 2,743 cases of environmental conflicts (n= 2,743). For some charts, only a subset of data from the same sample was analyzed. This is, for example, the case for comparisons across world income regions, because income information was not available for all countries.

Environmental conflicts analyzed in this study are largely recent environmental conflicts. 95% of the 2,743 cases analyzed here started during or after 1970; 50% of all cases started during or after 2008 and reach the present. We assume this temporal coverage to be the results of several factors: i) the EJAtlas was created in 2011 and has largely focused on recent cases; ii) arguably more secondary sources documenting environmental conflicts have become available over the last decades due to the growth in internet media such as online news, conflict databases, repositories of civil society reports, etc., and iii) a potential increase in environmental conflicts may have occurred globally over the last decade. The latter is difficult to assess as the number of total environmental conflicts globally is unknown. An overall increase in environmental conflicts is however likely, given that increases have been documented in relation to particular sectors (e.g. land conflicts related to land grabbing (Borras et al., 2011; GRAIN, 2008)), and specific concerns (e.g. killings of environmental defenders (Butt et al., 2019; Global Witness, 2014)).

The uneven geographical coverage, resulting from constraint access to information for some regions, limits possibilities for country comparisons. However, global income regions are relatively homogenously covered in the EJAtlas in terms of numbers of documented conflicts per millions of people (supplementary table 1). Here we compare regions only in terms of global income groups as defined by the World Bank.

Supplementary table 1. Coverage of environmental conflicts in the EJAtlas across world income regions.

Income regions	*Aggregated population (millions of people, 2010 values)*	*No. of conflicts reported in EJAtlas*	*No. of conflicts per millions of people*
Low-income countries	612.27	293	0.48
Lower middle	2,681.26	824	0.31
Upper middle	2,440.54	1,080	0.44
High-income countries	1,198.79	540	0.45

Source: Own elaboration based on EJAtlas (conflict data), and World Bank (2010 population data).

[1] The EJAtlas was created in 2011 with funding from the European Union (EU) project EJOLT. Two major follow-up research projects, the International Social Science Council's (ISSC) Transformations to Sustainability programme grant "ACKnowl-EJ" and the European Research Commission (ERC) advanced grant "EnvJustice", have further supported the development of the EJAtlas. Today it constitutes the largest database on environmental conflicts worldwide.

1.2 Other data sources

Country income data and classifications into low-income (LI), lower middle income (LM), upper middle income (UM), and high-income (HI) countries were retrieved from the World Bank's data catalog. Values for the 2010 calendar year (referring to World Bank's 2012 fiscal year, from July 1, 2011 to June 30, 2012) were used for all countries to group them into LI, LM, UM, and HI groups. Thresholds according to World Bank are as follows (values in Gross National Income per capita in US$, Atlas methodology): LI: <= 1,005; LM: 1,006 – 3,975; UM: 3,976 – 12,275; HI: > 12,275). Countries for which no income data were available have been excluded from those analyses employing country groupings per income status.

1.3 Data analysis software

Data were retrieved from the EJAtlas platform and have been cleaned, prepared, and analyzed with the Software *R Studio: A language and environment for statistical computing* (https://www.R-project.org/) (Version 1.1.456). Charts were created using Microsoft Excel, version Microsoft Office 365 ProPlus.

2 SUPPLEMENTARY RESULTS

This section shows the data tables that underlie the charts presented in the main article, as well as detailed results of the performed Pearson's Chi-square tests of independence. All data, if not stated otherwise, are taken from the EJAtlas dataset.

Supplementary table 2: Reported cases per type of environmental conflict (1st level category) (Figure 1b)

Conflict category (1st level classification)	*Number of cases*	*Percentage of total*
Mining	573	21%
Energy and climate	480	17%
Biomass and land use	422	15%
Water Management	379	14%
Infrastructure	248	9%
Industrial	213	8%
Waste Management	167	6%
Nuclear	100	4%
Conservation	98	4%
Tourism	63	2%

Total observations in table: n=2,743.

Supplementary table 3: Contingency table of occurrence of assassinations in relation to the main conflict category.

Conflict category (1st level classification)	*No assassinations*	*Assassinations*	*Total number of cases*
Biomass and Land	339 (80.33%)	83 (19.67%)	422
Conservation	86 (87.76%)	12 (12.24%)	98
Energy and Climate	438 (91.25%)	42 (8.75%)	480
Industries	189 (88.73%)	24 (11.27%)	213
Infrastructure	236 (95.16%)	12 (4.84%)	248
Mining	460 (80.28%)	113 (19.72%)	573
Nuclear	94 (94%)	6 (6%)	100
Tourism	60 (95.24%)	3 (4.76%)	63
Waste management	156 (93.4%)	11 (6.6%)	167
Water management	333 (87.86%)	46 (12.14%)	379

Total observations in table: n=2,743. Data are presented as frequency of cases and as percentages (in brackets).

Pearson's Chi-squared test results:

Chi^2 = 77.57907 d.f. = 9 p = 4.883084e-13

Minimum expected frequency: 8.084579

Supplementary table 4: Distribution of rural, semi-urban and urban conflicts across country income groups (n=2,737).

Income group	*Rural*	*Semi-urban*	*Urban*	*Not specified*	*Total number of cases*
High-income countries	217 (40.19%)	153 (28.33%)	134 (24.81%)	36 (6.67%)	540
Upper middle	637 (58.98%)	237 (21.94%	168 (15.56%)	38 (3.52%)	1080
Lower middle	591 (71.72%)	124 (15.05%)	76 (9.22%)	33 (4%)	824
Low-income countries	226 (77.13%)	40 (13.65%)	20 (6.83%)	7 (2.39%)	293

Data are presented as frequency of cases and as percentages (in brackets).

Supplementary table 5: Distribution of conflict types (1st level categories) across country income groups (Figure 2) (n=2,737).

Income group	*Conserv-ation*	*Biomass and Land*	*Energy and Climate*	*Industri-es*	*Infrastr-ucture*	*Mining*	*Nuclear*	*Tourism*	*Waste manag.*	*Water manag.*	*Total no. of cases*
High-income countries	18 (3.33%)	43 (7.96%)	124 (22.96%)	59 (10.93%)	76 (14.07%)	52 (9.63%)	56 (10.37%)	23 (4.26%)	44 (8.15%)	45 (8.33%)	540
Upper middle	29 (2.69%)	148 (13.70%)	147 (13.61%)	89 (8.24%)	76 (7.04%)	325 (30.09%)	21 (1.94%)	21 (1.94%)	76 (7.04%)	148 (13.70%)	1080
Lower middle	27 (3.28%)	135 (16.38%)	169 (20.51%)	55 (6.67%)	74 (8.98%)	143 (17.35%)	18 (2.18%)	16 (1.94%)	38 (4.61%)	149 (18.08%)	824
Low-income countries	24 (8.19%)	92 (31.40%)	39 (13.31%)	10 (3.41%)	22 (7.51%)	52 (17.75%)	5 (1.71%)	3 (1.02%)	9 (3.07%)	37 (12.63%)	293

Data are presented as frequency of cases and as percentages (in brackets).

https://doi.org/10.1016/j.gloenvcha.2020.102104

Supplementary table 6: Contingency table of occurrence of project cancellation in relation to diversification of protest (Figure 5)

Diversity of mobilization forms	*Project not canceled*	*Project canceled*	*Total number of cases*
Not diverse mobilizations (less than 5 mobilization forms used)	837 (92.79%)	65 (7.21%)	902
Diverse mobilizations (between 5 and 9 mobilization forms used)	1,216 (87.92%)	167 (12.08%)	1,383
Highly diverse mobilizations (10 or more mobilization forms used)	384 (83.84%)	74 (16.16%)	458

Total observations in table: n=2,743. Data are presented as frequency of cases and as percentages (in brackets).

Pearson's Chi-squared test results:
Chi^2 = 26.93526 d.f. = 2 p = 1.41606e-06
Minimum expected frequency: 51.09296

Supplementary table 7: Contingency table of occurrence of project cancellation in relation to mobilization start (preventive strategy) (Figure 5)

Mobilization start	*Project not canceled*	*Project canceled*	*Total number of cases*
Preventive mobilizations	836 (82.69%)	175 (17.31%)	1,011
Reactive mobilizations	925 (91.49%)	86 (8.51%)	1,011
Reparative mobilizations	474 (92.76%)	37 (7.24%)	511

Data are presented as frequency of cases and as percentages (in brackets). Note: For the analysis of the relation of project cancellation to mobilization start, the database was reduced to cases with preventive, reactive, and mobilizations for reparations. Latent cases and cases with unknown mobilization start have not been included. For definitions of categories, see supplementary table 14. Total observations in table: n=2,533.

Pearson's Chi-squared test results:
Chi^2 = 50.3588 d.f. = 2 p = 1.160717e-11
Minimum expected frequency: 60.11765

Supplementary table 8: Contingency table of occurrence of project cancellation in relation to legal actions (Figure 5)

Legal strategy	*Project not canceled*	*Project canceled*	*Total number of cases*
Lawsuits and EIA objections	350 (84.54%)	64 (15.46%)	414
EIA objections, no lawsuits	268 (87.87%)	37 (12.13%)	305
lawsuits, no EIA objections	713 (88.46%)	93 (11.54%)	806
No lawsuits, no EIA objections	1,106 (90.80%)	112 (9.20%)	1,218

Data are presented as frequency of cases and as percentages (in brackets). Total observations in table: n=2,743.

Pearson's Chi-squared test results:
Chi^2 = 12.86948 d.f. = 3 p = 0.004927538
Minimum expected frequency: 34.02479

Supplementary table 9: Contingency table of occurrence of project cancellation in relation to combined mobilization strategies (Figure 5)

Strategy	*Project not canceled*	*Project canceled*	*Total number of cases*
Combined strategy (cases with preventive and highly diverse mobilizations, as well as judicial activism and EIA objections)	74 (73.27%)	27 (26.73%)	101
All other cases	2,363 (89.44%)	279 (10.56%)	2,642

Data are presented as frequency of cases and as percentages (in brackets). Total observations in table: n=2,743.

Pearson's Chi-squared test results:
Chi^2 = 25.67183 d.f. = 1 p = 4.046905e-07
Minimum expected frequency: 11.26723

Supplementary table 10: Key positive and negative conflict outcomes (n=2,743) (Figure 6a).

Outcomes	*Number of cases*	*As percentage of total number of cases*
Strengthening of participation	798	29%
Environmental improvements	341	12%
Project canceled	306	11%
Negotiated alternative solutions	280	10%
Successful court decisions	454	34%
Failed court decisions	259	18%
Displacement and migration	571	21%
Criminalization of activists	547	20%
Violence against activists	503	18%
Assassinations	352	13%

n=2,743, except for successful and failed court decisions, where n=1,220.

Supplementary table 11: Contingency table of occurrence of death through murder in relation to involvement of Indigenous people in mobilizations (Figure 6b)

Involvement of Indigenous people	*No assassinations reported*	*Assassinations reported*	*Total number of cases*
Indigenous groups and local communities not involved in mobilizations	1,475 (91.61%)	135 (8.39%	1,610
Indigenous groups and local communities involved in mobilizations	916 (80.85%)	217 (19.15%)	1,113

Total observations in table: n= 2,743. Data are presented as frequency of cases and as percentages (in brackets).

Pearson's Chi-squared test results:
Chi^2 = 68.92822 d.f. = 1 p = 1.02113e-16
Minimum expected frequency: 145.3941

Supplementary table 12: Contingency table of occurrence of violent targeting of activists in relation to involvement of Indigenous people in mobilizations (Figure 6b)

Involvement of Indigenous people	*No violence against activists reported*	*Violence against activists reported*	*Total number of cases*
Indigenous groups and local communities not involved in mobilizations	1,395 (86.65%)	215 (13.35%)	1,610
Indigenous groups and local communities involved in mobilizations	845 (74.58%)	288 (25.42%)	1,113

Total observations in table: n=2,743. Data are presented as frequency of cases and as percentages (in brackets).

Pearson's Chi-squared test results:

Chi^2 = 64.645 d.f. = 1 p = 8.968381e-16

Minimum expected frequency: 207.7649

Supplementary table 13: Contingency table of occurrence of criminalization in relation to involvement of Indigenous people in mobilizations (Figure 6b)

Involvement of Indigenous people	*No criminalization reported*	*Criminalization reported*	*Total number of cases*
Indigenous groups and local communities not involved in mobilizations	1,368 (84.97%)	242 (15.03%)	1,610
Indigenous groups and local communities involved in mobilizations	828 (73.08%)	305 (26.92%)	1,113

Total observations in table: n=2,743. Data are presented as frequency of cases and as percentages (in brackets).

Pearson's Chi-squared test results:

Chi^2 = 58.87424 d.f. = 1 p = 1.680786e-14

Minimum expected frequency: 225.9391

3 SUPPLEMENTARY DEFINITIONS

Supplementary table 14: Definition of variables, terms and categories used in the EJAtlas and the main article.

Variable group	*Full name (as appearing on www.ejatlas.org)*	*Short name*	*Definition and description of the variable/term/category*
Main conflict category (1st level classification)	Biodiversity conservation conflicts	Conservation	Conflicts involving terrestrial and aquatic nature conservation initiatives. Examples: natural parks and other conservation zones, REDD+, wildlife corridors, etc.
	Biomass and land conflicts (Forests, agriculture, fisheries and livestock management)	Biomass and land use	Conflicts involving land uses for agriculture, forestry, livestock or fisheries. Examples: land acquisition for agribusiness, tree plantations, livestock farms, aquaculture, timber logging, extraction of non-timber products, etc.
	Fossil fuels and climate justice/energy	Energy and climate	Conflicts involving energy production or conflicts caused by climate change. Examples: oil and gas exploration, extraction or refining, oil spills, coal extraction or processing, gas flaring, fracking, solar or wind energy mega-projects, geothermal energy installations, climate-change-related conflicts such as the disappearance of glaciers or islands, etc.
	Industrial and utilities conflicts	Industries	Conflicts involving industrial activities and related pollution. Examples: chemical industries, metal refineries, manufacturing activities, cement or aluminum factories, industrial zones, etc. It also includes military installations and related pollution.
	Infrastructure and built environment	Infrastructures	Conflicts involving the construction of infrastructures, such as urban development projects, transportation infrastructures, as well as to the pollution resulting from them. Examples: ports, airports, highways, roads, railways, pipelines, canals, housing plots, etc.
	Mineral ores and building materials extraction	Mining	Conflicts involving mining, including extraction, transportation, waste material disposal, raw processing. Examples: conflicts linked to mineral ore exploration and processing, tailings from mines, extraction of building materials such as sand, gravel, quarries, etc.
	Nuclear	Nuclear	Conflicts involving nuclear energy production and waste disposal, including uranium extraction, transportation, nuclear power plants, nuclear waste storage, etc.
	Tourism recreation	Tourism	Conflicts involving the construction of tourism facilities or the establishment of exclusive recreational areas that are mainly used as touristic attractions. Examples: hotels or marinas, fun parks, ski resorts, enclosure of coastal areas for mass tourism, urban speculation, cruises or exclusive touristic airports, etc.
	Waste management	Waste management	Conflicts involving waste management, either creating unequal environmental burdens and/or health problems in specific areas, or over privatization of waste. Examples include incinerators, co-incineration facilities, toxic and e-waste disposal, polluting landfills, uncontrolled or

			unwanted dumping sites, ship-breaking yards, waste privatization, excluding waste pickers from their recycling practices etc.
	Water management	Water management	Conflicts linked to the access to and control over (mainly fresh-) water resources. Examples include dams, transboundary water conflicts, inter-basin water transfers, desalination plants, water access rights and entitlements, water privatization, water treatment and access to sewage facilities, etc.
Mobilizing groups (actors)	Waste pickers, informal recyclers	Waste pickers, recyclers	People that collect and segregate recyclable waste, in order to sell the materials to make a livelihood.
	Artisanal miners	Artisanal miners	Informal workers extracting minerals as a source of subsistence.
	Pastoralists	Pastoralists	Farmers dedicated to animal husbandry, normally moving herds in search of fresh pastures and water.
	Industrial workers	Industrial workers	Workers in different types of industries, including oil, mineral, chemical and waste facilities, as well as in infrastructure buildings such as airports, highways and industrial corridors, etc.
	Informal workers	Informal workers	People performing different types of informal labor, often not registered or regulated. Examples include construction workers, plantation seasonal workers, etc.
	Recreational users	Recreational users	Groups mobilizing for recreational activities and spaces of cultural, ecological and aesthetic importance which are at risk due to a contentious project. Examples include promoters of water sports on rivers vs. dam industries, supporters of ecological tourism vs forest industry, etc.
	Landless peasants	Landless peasants	People who do not have any land for farming or are prevented from owning the land they farm.
	Trade unions	Trade unions	Formal or institutionalized organization of workers who coordinate to achieve common interests regarding their working conditions (safety standards, wages, social security, etc.).
	Religious groups	Religious groups	Spiritual or religious institutions, religious movements (e.g. liberation theology groups, Buddhist monks, etc.) and individuals mobilizing through their spiritual beliefs against projects affecting their sacred grounds, activities, values, etc.
	Ethnically/racially discriminated communities	Discriminated groups	Group defined by their ethnic or racial status discriminated in their region due to misrecognition by the government, by old and new forms of colonialism, or due to general social discrimination. Examples include Roma communities, Latino, afro or migrant collectives in big cities, etc.
	Women	Women collectives	Women collectives or women organizations playing a key role in the mobilization against the contentious activity, either because they are affected by specific impacts (health, labor, household conditions, sexual exploitation, discrimination, or murder), or because they lead the main narratives of resistance and transformation.

	Fisher people	Fisherfolks	Fisher communities, who obtain their main source of nutrition and/or income from fishing (both from marine and freshwater areas), and small-scale commercial fishers usually organized through cooperatives or associations.
	International EJOs	Environmental justice organizations (international)	Transnational civil society organizations supporting resistance and counter-knowledge production in conflicts over resource extraction or waste disposal. They have an international profile (scope and influence beyond national borders) and include NGOs, coalitions, formal and informal activist networks, etc.
	Government, political parties	Government and political parties	Actors within different levels of government (executive, legislative and judicial powers at national or local level, political parties and government bodies) involved in a specific environmental conflict, by supporting claims of affected populations.
	Social movements	Social movements	Networks of a plurality of individuals, groups and/or organizations that recognize themselves as part of one movement, operating on the basis of shared collective identities or scopes. They may have existed before the conflict event or formed as a response to it.
	Local scientists/professionals	Scientists and professionals	Individuals or collectives providing professional, scientific, and technical knowledge to support claims for environmental justice. Some examples include toxicological evidence of health risks or independent assessments regarding social and environmental impacts.
	Indigenous groups or traditional communities	Indigenous groups or traditional communities	Communities and ethnic groups that recognize themselves as indigenous, tribal or traditional. Indigenous and tribal peoples are often known by national terms such as native peoples, aboriginal peoples, first nations, Adivasi, etc. Traditional communities include afro-descendent communities, such as quilombos, Garifuna, etc.
	Farmers	Farmers	People performing agriculture, raising field crops for food or raw materials, as well as livestock. This category refers to farmers who own and work on their own land. It includes both small-scale subsistence farmers, as well as larger landowners.
	Neighbors, citizens, communities	Neighbors	Urban and rural community members defined by proximity or common interest for an EJ cause and mobilizing together against a specific project that affects their immediate environment or interest. They include people not necessarily organized into formal collectives or associations.
	Local EJOs	Environmental justice organizations (local)	Civil society organizations or informal collectives involved in the conflicts at a local scale. They frequently have a local profile when their scope and influence focus on a specific territory, or can act on the country level. They include NGOs, associations and other grassroots organizations.
		Others	This category includes all other forms of mobilization not included above. If checked, the contributors should explain.
Mobilization forms (actions)	Appeals/recourse to economic valuation of the environment	Appeals to economic valuation	Arguments and methods valuing nature in economic terms as a means to defend against its destruction. This may include 'natural capital accounting', 'ecosystem services' and 'biodiversity offsets'.

	Street protests	Street protests	A protest march is a type of protest or demonstration that generally involves a group of people walking from an assembly point to a predetermined destination, usually culminating in a political rally.
	Development of a collective action/network	Collective action	Creation of a collective subject and first organized actions that did not exist before the specific conflict, often in terms of a committee, an association or even a network. These can be at local, national or transnational level.
	Blockades	Blockades	Acts or means of sealing off a place to prevent goods or people from entering or leaving, often preventing access to a disputed area.
	Involvement of (inter)national NGOs	Involvement of NGOs	The involvement of national and international NGOs to strengthen, support, increase outreach, or integrate the resistance into a broader overarching theme and agenda.
	Media-based activism	Media-based activism	Massive use of different forms of media- be it print or online-, as well as social media as one of the main strategies of the mobilization. A few examples could be video clips, alternative media channels, viral songs and videos in support of the communities' protests etc.
	Arguments for the right of nature	Rights of nature argument	Mobilization arguing that nature is not merely a property to be owned, but rather an entity which has an independent right to exist and flourish.
	Occupation of buildings/public spaces	Occupation of public spaces	Occupation of public spaces or buildings, which could include public offices, such as district headquarters or offices of the involved companies. In these cases, people protest by physically placing themselves, or other material, on strategic spots to make their demands heard.
	Land occupation	Land occupation	Protestors physically occupy an area of land for an extended period, frequently sleeping and living there. Such occupations usually take place on the site of a conflictive project or contested land, to physically prevent the project from taking off and/or continuing while engaging in forms of alternative living and collectivity building.
	Artistic and creative actions	Artistic actions	Use of creative ways, art and humor to draw attention to environmental conflicts. It can range from guerilla theatre, street plays, fairs and parties, music, puppet shows to murals, graffiti, banners, etc.
	Hunger strikes and self-immolation	Self-sacrifice	Bodily harming oneself, by hunger strike or other forms of self-immolation. It brings across the message that if a particular project is carried forward, then the only option left for a person or community would be to perish.
	Boycotts of companies/products	Boycotts of companies/products	Collective action against or ban of a company, by not purchasing or using its products. It aims to diminish economic performance of conflictive companies, or/and generate awareness about a company's policies, plans or industries which are causing environmental injustices
	Official complaint letters and petitions	Formal petitions	Official or public complaint letters which are often directed at government bodies, financial actors and banks, or companies, as well as online and offline petitions to collect signatures against a specific project.

	Lawsuits, court cases, judicial activism	Lawsuits	Legal actions using the existing environmental laws and regulations, which can happen at local or national fora level, or international courts. It includes collective or individual legal initiatives.
	Objections to EIAs	EIA objections	Formal objections to the Environmental Impact Assessment (EIA) through official, legal, procedural or administrative channels.
	Strikes	Strikes	Refusal to work or carry out a certain activity, performed in an organized fashion, typically used in an attempt to gain concessions from their employer or from the government.
	Referendum and other local consultations	Referenda	This refers to both regulatory or consultation process in which the public is asked to provide input in matters which directly affect them through official and publicly recognized procedures (sometimes even binding) as well as community organized consultations. This category includes both officially recognized and self-organized consultations, both at local and national level.
	Boycotts of official procedures	Boycotts of official processes	Intentional non-cooperation and non-participation in official procedures, such as public hearings or official consultations.
	Refusal of compensation	Refusal of compensation	Rejection of the form or criteria for assessing reparations, or directly rejecting money offered as compensation.
	Shareholder/financial activism	Financial activism	Actions by shareholders to support claims of affected groups. For example, buying shares in a publicly traded company that is causing environmental injustices so as to be able to attend the stakeholders' meetings and influence decision making.
	Creation of alternative reports, knowledge	Alternative knowledge creation	Production of new and alternative knowledge by activists, communities, or scientists, to challenge information produced by the state or companies. It includes performing alternative studies and preparing reports based on local knowledge and wisdom.
	Development of alternative proposals	Alternative proposals	Proposals for alternatives to the contentious project. It includes alternative proposals from communities' traditional knowledge and/or participative research with other collectives. It can be issued in the form of a formal report or through narrative claims-making.
	Community-based participative research	Participative research	Collection and analysis of data, performed by the communities themselves, or in collaboration with supportive organizations or committed researchers. Examples include popular epidemiology instances on local health and the local environment, forming coalitions with scientists and building citizen–scientist collaborations to advance their claims and seek legitimacy for their views.
	Property damage	Property damage	Physically targeting objects, infrastructures or buildings, as symbol of the causes of environmental injustices. It includes setting property or objects on fire, breaking or vandalizing houses and offices, puncturing tires of vehicles, burning effigies etc.
	Sabotage	Sabotage	Destruction or vandalism of property, often to prevent any project to begin or continue operating. An example of sabotage could be destruction of fences or walls created for enclosing common land.

https://doi.org/10.1016/j.gloenvcha.2020.102104

	Threats to use arms	Threats to use arms	Non-peaceful and potentially violent forms of mobilization. It refers to the use of arms and ammunitions, be it guns and grenades, or even locally used arms such as bows and arrows, axes, etc.
	Public campaigns	Public campaigns	Actions aiming at generating awareness about the harmful impacts of certain projects or the destructive policies of certain companies involved in environmental conflicts. They can be of local, national or transnational nature, formally led by a set of organizations and promoters or animated by general slogans that are later picked up locally in multiple forms and narratives.
		Others	All other forms of mobilization not included above. If checked, the contributors should explain.
Outcomes	Deaths, Assassinations, Murders	Assassinations	Death of one or more protestors, intentionally caused by a third party. Death can occur on the spot, for example when shooting to death environmental defenders, or be caused following wounds, rapes, tortures, etc.
	Criminalization of activists	Criminalization	It includes criminal prosecutions of individuals and abuses of civil and human rights, the opening of criminal investigations unlikely to reach the trial stage used to disarticulate, demoralize and discourage social protest, and the use of disproportionate sentences for offenses to punish practices often deployed in social protests.
	Repression	Repression	Threat to subdue or act of subduing protests by institutional or physical force. Includes a variety of tactics (frequently including violent and coercive actions, violating rights) taken by government, or security staff, militias or corporate actors, to quell dissent and protests.
	Violent targeting of activists	Violence against activists	Physical harassment, injuring or assassinations of specific targeted persons, usually key activists, or to implant fear to defer environmental defenders' actions. Examples include violent threats to activists and their relatives, death threats, sexual threats, accident attempts, etc.
	Strengthening of participation	Strengthening of participation	Increased civic engagement and public participation in consultation, planning and politics as an outcome of mobilization and collective organizing in the conflict.
	Project canceled	Project canceled	The contested project, or activity, is stopped. The decision can be made by the government or the company itself, both in initial, or later stages. The decision is usually confirmed in official documents or announcements. However, it does not necessarily mean that the conflict is over, nor that impacts are not there anymore.
	Environmental improvements, rehabilitation/restoration of area	Environmental improvements	Reparations, interventions or restoration of the environment so as to improve the ecological conditions of the area.
	Court decision (victory for environmental justice)	Successful court decisions	Cases where legal action is taken, and the courts rule in favor of the environmental defenders. Such legal victories can include for example cancellation of projects, orders for creating better regulations, total ban on the conflictive activity or for a stipulated period, orders for re-evaluation of impact assessments, compensation demands, etc.

	Court decision (undecided)	Undecided court decisions	Cases where legal action has been taken but no clear decision has yet been issued or the case is ongoing.
	Court decision (failure for environmental justice)	Failed court decisions	Cases where legal action has been taken but the ruling is in favor of the industries or the projects which triggered the conflict, or cases that dismissed the claims of affected people.
	Negotiated alternative solution	Negotiated alternative solution	Cases where some project parameters are modified, or where some affected groups gain benefits, through processes of negotiation with involved actors such as companies, governments and project authorities. Such negotiated solutions do not necessarily meet the demands and claims raised by all.
	Application of existing regulations	Application of existing regulations	Cases in which regulations that already existed before the conflict but were not being applied, are applied following court order, government action or as a result of public pressure.
	Compensation	Compensation	Compensation by the state or company to address loss and impacts related to the project, either through financial transfers or through in-kind compensations (goods and services aiming to amend the impacts suffered).
	Corruption	Corruption	Abuse of entrusted power for private financial or political gain. Here, this category captures those cases where corruption has been proven and/or condemned by a court judgment or evidenced and documented by mobilizing groups.
	Fostering a culture of peace	Fostering a culture of peace	Despite the conflict, communities and mobilizing groups have worked for peaceful resistance and for fostering a culture of peace.
	Institutional changes	Institutional changes	Cases where the conflictive situation has brought to changes in the institutional systems, for example by setting up government bodies for monitoring impacts, or new local authority systems.
	Land demarcation	Land demarcation	Demarcation of lands is the formal process of identifying the actual locations and boundaries of Indigenous lands or territories or more broadly of clarifying territorial boundaries.
	Migration/displacement	Migration and displacement	Forced or otherwise induced movement of peoples due to the conflictive project or activity. It includes displacement according to resettlement programs or without any such scheme. It can be a direct impact of the conflictive project or an indirect, gradual consequence of it across time.
	Moratoria	Moratoria	Temporary or indefinite suspension of the conflictive activity beyond a specific project, through legal tools. Moratoria delay in time the contentious activity but do not necessarily ban it over longer periods. It includes moratoria to future projects, or specific aspects and practices of current activities. Examples are provincial or countrywide bans on timber logging, large-scale agricultural concessions, fossil fuel extraction, open cast mining, GMOs, approval of new nuclear power plants, etc.

	New Environmental Impact Assessment/Study	New Environmental Impact Assessment/Study	Cases in which, as a result of the mobilizations, public authorities decide to conduct a new environmental impact assessment (EIA) of the proposed investment project. It includes both cases where there were no initial EIAs at all, and those where an environmental impact study was previously submitted but was deemed incomplete or flawed.
	New legislation	New legislation	Cases in which, as a result of mobilizations and political debates around them, public authorities issue new legislation. It includes new legislation either in favor or against the mobilized groups.
	Project temporarily suspended	Project temporarily suspended	Suspension of the contentious project due to protests and claims, or for financial and political reasons. The suspension can last days, months or even years. During the suspension, the project can be re-negotiated, and/or another EIA can be carried out, or the project can be finally scrapped.
	Technical solutions to improve resource supply/quality/distribution	Technical solutions	It refers to technical solutions to minimize impacts or the number of affected people and land—for example, new filters in incinerations to reduce emissions.
	Under negotiation	Under negotiation	Cases where a process of dialogue or negotiation is in place among some of the actors involved in the conflict. This negotiation can take place while the contentious project is planned, operating, in construction, suspended.
	Withdrawal of company/investment	Withdrawal of company or investment	Cases where companies involved in the conflict withdraw or stop their investment. Reasons for their withdrawal could include economic viability, internal business decisions, pressure from the environmental movement, etc. However, it does not necessarily imply the suspension of the contentious project, as other companies or investors can get involved.
	Other		All other types of outcomes not included above. If checked, the contributors should explain.
Mobilization start	LATENT (no visible resistance)	Latent	A latent conflict is one without visible resistance and mobilizations, but where there are reasonable signals that it might appear anytime.
	PREVENTIVE resistance (precautionary phase)	Preventive mobilizations	Conflicts in which mobilization begins before the project begins to be operated or constructed. Examples include those cases when mobilizations begin during public consultation processes.
	In REACTION to the implementation (during construction or operation)	Reactive mobilizations	Conflicts in which mobilizations start after the construction or operation of a contentious project.
	Mobilization for reparations once impacts have been felt	Reparative mobilizations	Cases in which the project already produced harm and caused adverse impacts to the environment and/or to local groups, and where mobilization begins once the impacts have been felt.
	UNKNOWN	Unknown	When the inception of the mobilization is not certain.
